# Psychological impact of COVID-19 pandemic on frontline health care workers in Bangladesh: A cross-sectional study

**DOI:** 10.1192/bjo.2021.621

**Published:** 2021-06-18

**Authors:** Suman Ahmed, Mohammad Shamsul Ahsan, Rubaiya Khan, Mahbubul Hasan, Fahmida Ferdous, Humayra Shahjahan, Murin Hossain, Ananya Kar, Kamrul Hossain

**Affiliations:** 1 Tees, Esk and Wear Valleys NHS Foundation Trust; 2 Bangabandhu Sheikh Mujib Medical University; 3 Daffodil International University

## Abstract

**Aims:**

Frontline health care workers exposed to COVID-19 patients could be at increased risk of developing psychological issues. The study aimed to estimate the prevalence of mental health-related problems, specifically depression, anxiety, post-traumatic stress disorder (PTSD), and insomnia among health care professionals during the COVID-19 pandemic in Bangladesh and to compare these between medical and allied health care professionals.

**Method:**

This cross-sectional survey was conducted using Google Form then subsequent telephone interview between June and August 2020. Using random sampling, a total of 479 health care professionals participated in the study. We collected data on demographics. Anxiety and depression were measured using 4 items Patient Health Questionnaire-4 (PHQ-4), PTSD was measured using 4 items Primary Care (PC)-PTSD-Screen, and insomnia was measured by using a 7-item Insomnia Severity Index (ISI). A multivariable logistic regression analysis was performed to assess risk factors associated with mental health symptoms.

**Result:**

Overall, 17.6% of frontline health workers had symptoms of anxiety, 15.5% had depression symptoms, 7.6% had PTSD symptoms and 5.9% had symptoms of insomnia. Compared to allied health professionals (n = 113, 24%), doctors (n = 366, 76%) had significantly higher prevalence of anxiety: 21.1% vs 06%, (OR = 4.19; 95% CI = 1.88–9.35; p-value <0.001); depression: 18% vs 6.8%, (OR = 2.99; 95% CI = 1.40–6.42; p-value 0.005); PTSD: 9.4% vs 1.7%, (OR = 5.96; 95% CI = 1.41–25.11; p-value 0.015) and insomnia: 7.4% vs 0.9%, (OR = 9.22; 95% CI = 1.24–68.4; p-value 0.03). Logistic regression analysis showed that pre-existing medical illness has significantly more risks of developing symptoms of anxiety (adjusted OR = 2.85; 95% CI = 1.71–4.76; p-value <0.001) and depression (OR = 2.29; 95% CI = 1.39–3.77; p-value 0.001). Having a postgraduate degree (adjusted OR = 6.13; 95% CI = 1.28–29.28; p-value 0.023) and working in secondary care setting (adjusted OR = 3.08; 95% CI = 1.18–8.02; p value 0.021) have significant predictors of developing anxiety symptoms among health workers. Those who had worked more than 6 weeks in COVID-19 dedicated hospitals had risk of developing symptoms of PSTD (OR = 2.83; 95% CI = 1.35–5.93; p value 0.006) and insomnia (OR = 2.63; 95% CI = 1.15–6.02; p value 0.022).

**Conclusion:**

Our study demonstrated a high prevalence of symptoms of depression, anxiety, PTSD, and insomnia among Bangladeshi frontline health workers (particularly among doctors) during the COVID-19 pandemic. There is an urgent need to address the mental health needs of frontline health workers.

Funding: Medical Research Council, Bangabandhu Sheikh Mujib Medical University (BSMMU), Dhaka, Bangladesh.

